# Transcriptomic Analysis Reveals the Molecular Relationship Between Common Respiratory Infections and Parkinson’s Disease

**DOI:** 10.3390/cimb47090727

**Published:** 2025-09-07

**Authors:** Abdulaziz Albeshri, Ahmed Bahieldin, Hani Mohammed Ali

**Affiliations:** Department of Biological Sciences, Faculty of Science, King Abdulaziz University, Jeddah 21589, Saudi Arabia; abmahmed@kau.edu.sa

**Keywords:** Parkinson’s disease, respiratory infections, COVID-19, influenza, bacterial pneumonia

## Abstract

Parkinson’s disease (PD) is one of the most rapidly growing neurological disorders globally. The molecular relationship between common respiratory infections (RIs) and idiopathic Parkinson’s disease (iPD) remains a controversial issue. Multiple studies have linked acute respiratory infections to PD, but the molecular mechanism behind this connection is not significantly defined. Therefore, the aim of our study was to investigate potential molecular interactions between RIs and PD. We retrieved eight publicly available RNA-seq datasets from the NCBI Gene Expression Omnibus (NCBI GEO) and performed extensive bioinformatics analysis, including differential gene expression (DGE) analysis, the identification of overlapped differentially expressed genes (DEGs), weighted gene co-expression network analysis (WGCNA), pathway and functional enrichment analysis, the construction of protein–protein networks, and the identification of hub genes. Additionally, we applied a machine learning method, a Random Forest model (RF), to external RIs datasets to identify the most important genes. We found that ribosomal subunits, mitochondrial complex proteins, proteasome subunits, and proteins encoding ubiquitin are simultaneously downregulated and co-expressed in RIs and PD. Dysregulation of these proteins may disturb multiple pathways, such as those responsible for ribosome biogenesis, protein synthesis, autophagy, and apoptosis; the ubiquitin–proteasome system (UPS); and the mitochondrial respiratory chain. These processes have been implicated in PD’s pathology, namely in the aggregation of α-synuclein, mitochondrial dysfunction, and the death of dopaminergic neuron cells. Our findings suggest that there are significant similarities in transcriptional responses and dysfunctional molecular mechanisms between RIs, PD, and aging. RIs may modulate PD-relevant pathways in an age- or immune-dependent manner; longitudinal studies are needed to examine the RIs risk factor. Therefore, future studies should experimentally investigate the influence of age, vaccination status, infection type, and severity to clarify the role of RIs in PD’s pathogenesis.

## 1. Introduction

Globally, PD has become the second most prevalent chronic disorder causing neurologic deterioration [1]. PD is identified by symptomatic signs including resting tremors, bradykinesia, rigidness, and postural instability, along with a range of signs indicating additional motor and non-motor impairment. With an aging global population and increases in life expectancy, age-associated disorders such as PD are gaining more attention in the field of neurological sciences. Neurological disorders currently represent the primary cause of disability globally, and PD is the most rapidly increasing neuro-disorder in terms of prevalence [2]. Even though PD is a non-infectious disease, the spread of PD has many hallmarks of a pandemic. Pandemics grow across wide geographical regions, and PD rates are rising in every major area of the world. Pandemics also tend to travel, and the incidence of PD, while growing worldwide, appears to be changing in response to increases in aging and urbanization [3]. Pathologically, PD results from the specific degradation of dopaminergic neurons (DNs) in the substantia nigra (SN), leading to reduced dopamine levels in the striatum and resulting in deteriorating motor control. The origin of PD remains largely unknown; however, studies indicate that it contains multiple risk factors, such as genetics variants, environmental variables, and aging [4]. Cellular pathways associated with mitochondrial function, misfolding protein accumulation, and neuroinflammation reveal that many vital mechanisms contribute to PD-related pathology, including α-synuclein aggregation and lysosomal dysfunction [5].

The hypothesis of an infectious origin of PD was first proposed several decades ago, particularly following the Spanish flu pandemic, when parkinsonian symptoms were observed in individuals infected with the influenza virus who later developed encephalitis lethargica [6]. Numerous epidemiological and pathological investigations have attempted to explore the association of infectious diseases with neurological disorders; nonetheless, the critical role of pathogenic microbes in neurodegenerative disorders remains under investigation [7]. Microbes may cause impairment of the nervous system through various mechanisms. Direct infection and reproduction can result in clinical syndromes such as encephalitis, myelitis, and meningitis [8]. Microbial infections can additionally activate post-infectious immune response in the entire nervous system, as observed in Guillain–Barré syndrome and immune-mediated demyelinating encephalomyelitis. Some viral infections can persist in an active or mutated form within the central nervous system (CNS) or peripheral ganglia, which may result in subsequent reactivation and neurological disease [9]. Neuroinflammation generally appears as an inflammatory reaction of the neuroimmune system induced by several damaging factors, including microbial infection, intracranial injuries, or toxins. This response is characterized by the production of various anti-inflammatory factors, nitric oxide, and reactive oxygen species by innate immunity in the CNS [10]. Signs of neurological inflammation in individuals with PD were first observed by McGeer and his colleagues [11]. All brain cell types, including neurons and glial cells, have the ability to initiate and respond to inflammatory responses during infections [12]. Neurotropism refers to the ability of microbes to invade the CNS [13]. Some viruses have displayed significant neurotropism, identified in post-mortem brain parenchyma, alongside other viruses that appear devoid of evident neurotropism yet induce neurological indications [14].

Considerable evidence has suggested involvement of RIs in the onset of PD. Olfactory receptor neurons are uniquely positioned at the interface with the external environment, where their dendritic terminals come into direct contact with airborne macromolecules. These molecules can be taken up by the neurons and subsequently transported to the CNS through transsynaptic pathways [15]. Olfactory loss is abundantly prevalent in individuals suffering from PD, representing an early and straightforward identifying factor, and it persists throughout the progression of the disorder. Olfactory dysfunction occurs in almost 90% of individuals with PD and serves as a possible preclinical biomarker, as well as a primary prodromal symptom that can precede neuropathology [16]. Braak and his colleagues reported that the olfactory bulb and anterior olfactory nucleus initiate the neuropathological process and are the earliest lesions in the brain of those of iPD besides the dorsal motor nucleus of the vagus nerve [17]. However, evaluation of olfactory deficits in population-based cohort studies has demonstrated that impaired olfactory is a significant indicator of PD risk, with follow-up periods extending up to 10 years [18]. Correspondingly, viruses infecting the upper respiratory tract are the predominant cause of persistent olfactory dysfunction in individuals without neurological disorders [19]. Upper respiratory infections can induce various kinds of histopathological changes in the olfactory epithelium, which can be associated with functional impairment [20]. Viruses that were absorbed by olfactory receptor cells were found to be either blocked by virus-induced cellular apoptosis pathways or capable of producing anti-apoptotic factors that prevent neuronal apoptosis, facilitating invasion of the brain [21].

Among hospitalized influenza patients, 4.9% developed post-infectious neurological complications, most commonly migraine (3.2%), neuropathy (3.6%), movement disorders (2.5%), stroke (2.4%), dementia (2.3%), and epilepsy (2.1%), underscoring the risk of subsequent neurological complications following influenza [22]. Influenza and pneumonia were significantly linked to five neurodegenerative diseases: Alzheimer’s disease, amyotrophic lateral sclerosis, dementia, PD, and vascular dementia [23]. H1N1 influenza selectively disrupts proteostasis, inducing α-synuclein and DISC1 aggregates in human dopaminergic cells and Rag knockout mice. Mechanistically, H1N1 blocks autophagosome formation and autophagic flux, and intranasal infection produces α-synuclein aggregates in neurons connected to the olfactory bulbs, suggesting virus-driven proteostasis defects can facilitate pathogenic assemblies and contribute to synucleinopathies [24]. Two studies have identified viral RNA of SARS-CoV-2 in the brain of COVID-19 patients [25,26]. PD and Parkinsonism-like symptoms have been linked to multiple cases of SARS-CoV-2 infection, and SARS-CoV-2 was found to possibly act as an environmental trigger for the onset of PD in genetically susceptible asymptomatic individuals [27,28,29,30,31]. Recent longitudinal imaging data from the UK Biobank demonstrated that SARS-CoV-2 infection is associated with reduced gray matter thickness, tissue damage in regions connected to the olfactory cortex, decreased global brain size, and cognitive decline, even in non-hospitalized cases [32].

Therefore, we performed standard transcriptomics analysis empowered with machine learning to reveal potential molecular mechanisms shared between RIs and PD.

## 2. Materials and Methods

### 2.1. Data Retrieval

We searched the NCBI Gene Expression Omnibus (GEO) repository [33] using the terms “Parkinson’s disease”, “COVID-19”, “Influenza Virus”, “Bacterial pneumonia” and “Blood”, with the “homo sapiens” organism as a filter. To produce productive, comprehensive, and insightful results, we implemented the following criteria for the datasets:

(1) The datasets contain control samples to compare with.
(2) Only blood samples were used because circulating mRNA molecules can originate from various tissues and organs, providing a systemic profile of gene expression.
(3) Due to the limited accuracy of DNA microarray, we only included RNA-seq datasets.
(4) We excluded datasets derived from samples treated with special clinical interventions.

Based on these criteria, we selected seven datasets to represent RIs and one dataset to represent PD (Table 1).

### 2.2. Data Quality Control

Principal Component Analysis (PCA) was performed to evaluate the quality of RNA-seq data. The read counts were processed using a variance-stabilizing transformation method utilizing the vst function from the DESeq2 package (1.44.0 version) [34]. The plotPCA function was used to produce two-dimensional scatter plots for transformed data, and ggplot2 was used to customize the appearance of the plots with PC1 on the x-axis and PC2 on the y-axis. PCA of the top 500 variable genes with highest row variance was carried out across all samples to identify outliers above the two standard deviations of PC1 and PC2.

### 2.3. Differential Gene Expression (DGE)

We conducted a differential expression analysis of individuals’ responses during RIs and PD against healthy controls using the R package DESeq2 (1.44.0 version) to extract differentially expressed genes (DEGs) [34]. We selected the Benjamini and Hochberg false discovery rate method for the adjustment of *p*-values [35]. The cutoff values of the log2 fold change ≥ 1 and the adjusted *p*-value ≤ 0.05 were considered as statistically significant. We used the InteractiVenn website to generate a Venn diagram to present overlapping DEGs [36].

### 2.4. Weighted Correlation Network Analysis (WGCNA)

The WGCNA algorithm in R was employed for the development of a weighted gene co-expression network and the categorization of representative module genes associated with diagnostic characteristics [37]. We used RIs and PD datasets to find co-expressed-related RI-PD modules. Primarily, PCA was utilized to eliminate outlier samples. Normalization of the data was performed using the variance-stabilizing transformation method of the DESeq2 package [34]. The final number of genes was reduced by removing lower-expressed genes to avoid noisy and inadequate outcomes. The soft-thresholding powers were determined using the “pickSoftThreshold” function to pick the optimal soft threshold power (from 1 to 30). Subsequent steps included network construction, carried out in a block-wise manner; calculating module eigengenes; estimating the topological overlap matrix (TOM); constructing modules; and performing dynamic branch cutting with a merging threshold of 0.15 to define co-expressed modules. Significant module correlations between the gene expression level and disease state were defined. Co-expressed genes were extracted from positively related controls and subjected to further bioinformatics analysis. The ggPlot2 (3.5.2 version) and CorLevelPlot (0.99.0 version) packages were used to visualize the included cluster dendrogram plot and module–trait correlation heatmap [38,39].

### 2.5. Protein-Protein Interactions (PPIs) and Hub Genes

The overlapped genes obtained from DGE and WGCNA were submitted to the STRING database (v12.0) (http://www.string-db.org/, accessed on 17 July 2025) [40] to construct a medium-confidence network with an interaction score > 0.4. We enabled all the active interconnection references including experiments, co-expression, databases, gene fusion, neighborhood, text mining, and co-occurrence. The biomolecular interaction network visualization software “Cytoscape” (3.10.3 version) was used to visualize the networks [41]. To identify hub genes in the generated networks, we used the Cytoscape plugin “CytoHubba” (0.1 version). CytoHubba provides eleven global and local-based statistical algorithms to score the impact of genes in a molecular network based on their network features. The local rank methods specifically consider the interaction between the gene and its direct neighbors; in contrast, the global rank methods score the interaction between the genes and the whole network [42].

### 2.6. Enrichment Analysis

We performed enrichment analysis using the ClusterProfiler R package (4.14.6 version) [43]. Gene Ontology (GO) enrichment was carried out across all three ontologies (biological process, cellular component, and molecular function) using the org.Hs.eg.db annotation package (3.20.0 version) [44]. Parameters included Benjamini–Hochberg correction for multiple comparisons (pAdjustMethod = “BH”), with thresholds set at *p*-value < 0.01 and q-value < 0.05. KEGG pathway enrichment was performed using the enrichKEGG function with human gene identifiers (organism = “hsa”), employing a *p*-value cutoff of 0.05, a q-value cutoff of 0.2, and minimum and maximum gene set sizes of 10 and 500. Both overlapped DEGs and genes from WGCNA-derived modules were used as input for enrichment analyses.

### 2.7. Machine Learning Model (Random Forest)

We selected six datasets representing RIs (3 COVID-19 datasets and 3 influenza datasets) to construct machine learning models that can identify key transcriptomic features and differentiate RIs patients from healthy controls. We applied a Random Forest classification model on normalized and batch-corrected transcriptomic profiles. TPM matrices from six separate blood RNA-seq datasets were first trimmed to their shared genes and log2 transformed. To minimize technical variability across datasets, batch effects were corrected using the ComBat function from the sva package (3.56.0 version) [45]. To reduce dimensionality and enhance model interpretability, the top 5000 most variable genes across all samples were selected based on variance. A Random Forest classifier was trained using the randomForest R package using gene expression features to identify the most discriminative genes [46]. Model performance was evaluated by ROC curve analysis, with area under the curve (AUC) being computed using the pROC package (1.18.5 version) [47]. Variable importance was assessed via the Mean Decrease in Gini, and the top 100 important genes were extracted. PCA and UMAP were performed on the expression profiles of the top 100 RF-top genes using the stats (4.4.1 version) and umap (0.2.10.0 version) packages [48,49]. The ggplot2 was used to generate PCA, UMAP, ROC, and variable importance plots [38].

## 3. Results

### 3.1. Study Workflow

**Figure 1:**
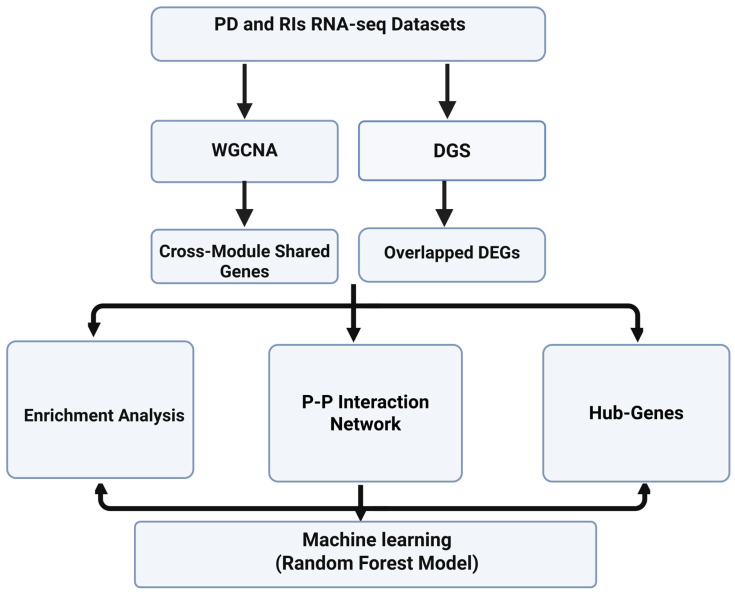
Analysis pipeline for RNA-seq datasets in PD and RIs studies. Figure is created using https://www.biorender.com.

The workflow of our study comprised seven steps, as illustrated in (Figure 1). First, we retrieved the RIs and PD blood tissue-based datasets from the NCBI GEO database. Two datasets were used for DGE and WGCNA, and others were employed to construct a machine learning model (Table 1). The GSE161731 datasets included data pertaining to seasonal coronavirus (*n* = 61), COVID-19 (*n* = 77), B. pneumonia (*n* = 24), influenza (*n* = 17), and controls (*n* = 19). The PD dataset, GSE161199, contains data pertaining to controls (*n* = 11) and PD (*n* = 5). Second, DEGs within each RIs condition (COVID-19, influenza, seasonal coronaviruses, B. pneumoniae) versus controls were extracted and then overlapped with the PD DEGs to identify genes shared across RIs and PD. Third, we performed WGCNA for the RIs and PD datasets to construct clusters of co-expressed genes and identify shared genes between the most trait-correlated modules. Fourth, we constructed protein–protein networks for both overlapping DEGs and shared co-expressed genes using the STRING database. Fifth, we identified hub genes using the Cytohubba plugin. Sixth, we performed enrichment analysis to determine enriched GO terms and KEGG pathways. Seventh, to extract the most important genes, we retrieved six RIs datasets, including influenza datasets (influenza: 111 samples; control: 52 samples) and COVID-19 datasets (COVID-19: 111 samples; control: 49 samples). We trained a Random Forest classifier and ranked genes by mean decrease in Gini (Gini importance) to identify the most discriminative genes for distinguishing RIs cases from controls.

### 3.2. Quality Control

**Figure 2:**
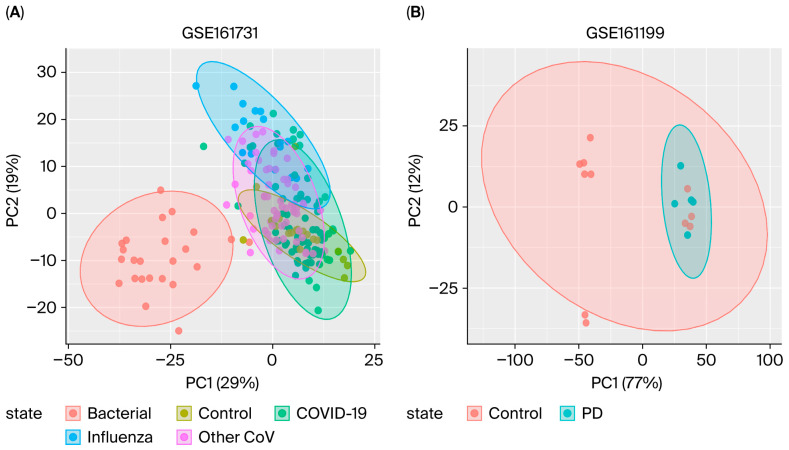
Principal component analysis (PCA) of transcriptomic datasets. (**A**) PCA plot of the GSE161731 dataset showing clustering of samples from bacterial infections (red), healthy controls (green), COVID-19 (turquoise), influenza (cyan), and other seasonal coronaviruses (purple). Each ellipse represents the 95% confidence interval for each group. The first two principal components (PC1 and PC2) explain 29% and 19% of the variance, respectively, indicating partial separation of bacterial from viral infections and controls. (**B**) PCA plot of the GSE161199 dataset comparing PD (cyan) and control (red) samples. PC1 accounts for 77% of the variance, and PC2 accounts for 12% of the variance, revealing a distinct clustering pattern between PD and controls.

We performed principal component analysis (PCA) to evaluate the clustering of RIs and PD samples based on their transcription patterns (Figure 2). In our PCA of RIs versus controls, samples from bacterial infections formed an intelligibly separated cluster along the first PCA (PC1, 29%), which discriminates them from viral infections and control samples. The COVID-19 and influenza samples exhibited partial overlap but were generally shifted relative to the control and seasonal coronaviruses groups along PC2 (19%), suggesting shared but also condition-specific transcriptional responses. Control samples formed a tighter cluster, indicating greater transcriptional homogeneity. In the PD versus control dataset, most PD and control samples formed distinct clusters, with a subset of control samples overlapping with PD cases, suggesting the presence of shared or transitional gene expression patterns. This partial overlap may reflect the increase in the baseline age regarding the control samples.

### 3.3. Differential Gene Expression and Overlapped DEGs

**Figure 3:**
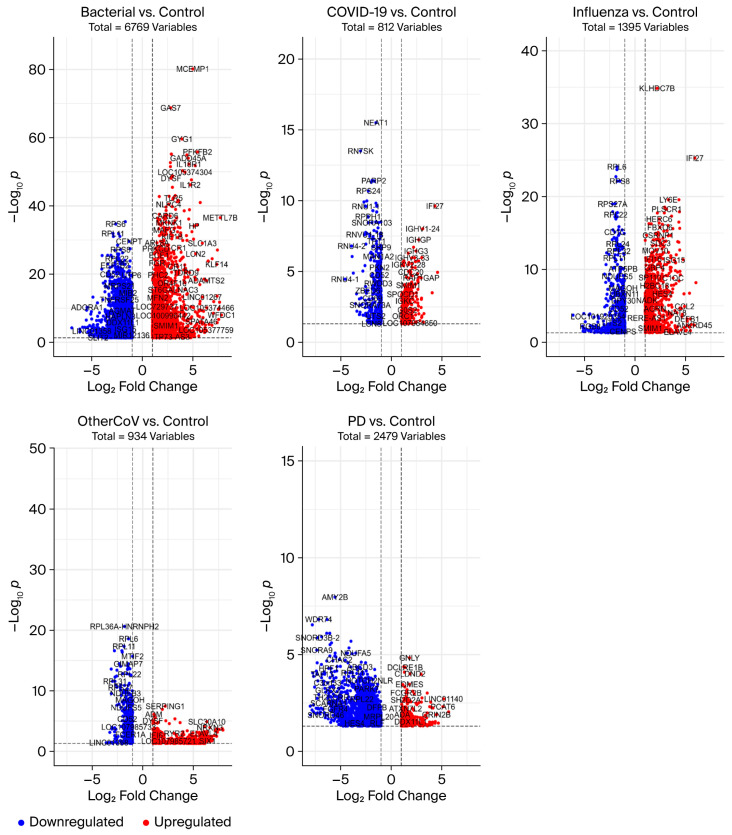
Volcano plots displaying the distribution of DEGs based on log2 fold change (x-axis) and statistical significance (−log10 *p*-value, y-axis) for each comparison. Red dots represent significantly upregulated genes, and blue dots represent significantly downregulated genes, as defined by adjusted *p* < 0.05 and |log2 fold change| ≥ 1. Labeled genes highlight representative transcripts with the strongest fold-change or statistical significance. Total numbers of DEGs per condition are indicated above each plot.

**Figure 4:**
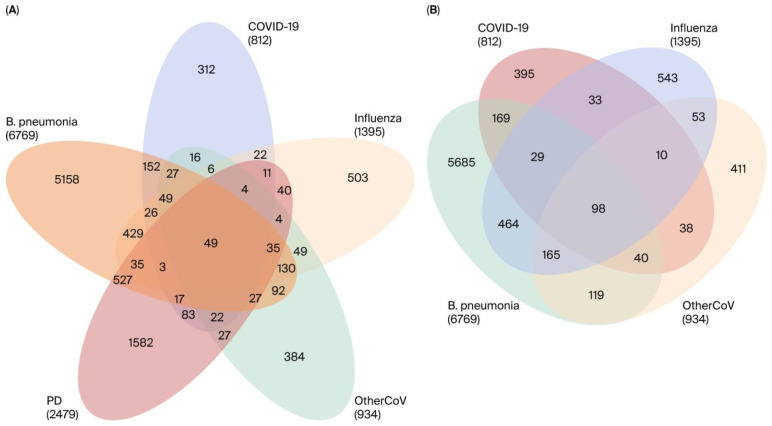
Overlap of DEGs across RIs and PD. (**A**) Quintuple Venn diagram showing shared and unique DEGs among bacterial pneumonia, COVID-19, influenza, other seasonal coronaviruses (OtherCoV), and PD. The numbers inside each intersection represent the count of overlapping DEGs between the respective conditions. Notably, 49 DEGs were common between PD and RIs. (**B**) Quadruple Venn diagram depicting DEG overlaps exclusively among RIs (bacterial pneumonia, COVID-19, influenza, and OtherCoV). A total of 98 DEGs were consistently dysregulated across all RIs, highlighting shared transcriptional responses to infection.

**Figure 5:**
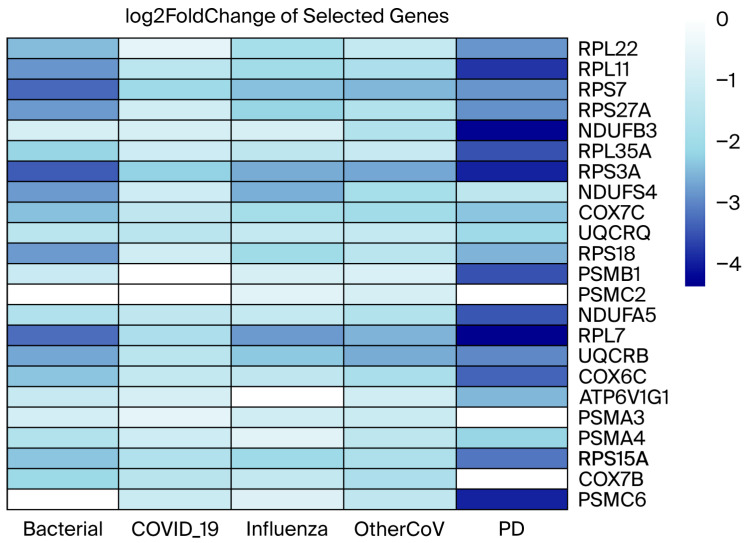
Heatmap of representative dysregulated genes highlighting shared transcriptional patterns in RIs and PD. The heatmap illustrates the log_2_ fold change of selected genes across bacterial pneumonia, COVID-19, influenza, other seasonal coronaviruses (OtherCoV), and PD. Genes included are representative members of three major categories consistently affected in both conditions: ribosomal proteins, mitochondrial respiratory complex subunits, and proteasome subunits. Blue shading indicates downregulation relative to controls, with darker colors reflecting stronger suppression. While not all genes are directly shared between RIs and PD, their collective dysregulation within these pathways highlights similar transcriptional signatures linking mitochondrial function, ribosomal activity, and proteasome regulation across both conditions.

To investigate the molecular interrelationship between RIs and PD, we analyzed publicly available RNA-seq datasets from the NCBI Gene Expression repository. Our aim was to identify genes that are differentially expressed across various types of RIs and assess their potential participation in PD-related molecular pathways (Figure 3). By classifying shared and condition-specific gene expression patterns, we sought to uncover key dysregulated genes that are shared across RIs and PD, which may provide insights into the transcriptional overlaps and possible molecular links between RIs and neurodegenerative processes in PD. We identified 934 DEGs in seasonal coronaviruses, 6769 DEGs in bacterial pneumonia, 1395 DEGs in influenza, and 812 DEGs in COVID-19, whereas in the PD dataset, we identified 2579 DEGs (Table 2). The Venn diagram shows that there are 98 DEGs that are commonly dysregulated across the RIs and 49 overlapping DEGs between RIs and PD (Figure 4); the complete sets of DEGs involved in both intra-RIs interactions and RIs–PD crosstalk are included in (Table 3). All the RIs–PD crosstalk genes are downregulated, except for the ribosomal protein L27 (RPL27), which was downregulated in RIs but not in PD. Notably, ribosomal proteins, mitochondrial respiratory complex proteins, and proteasome subunits were consistently downregulated, particularly in PD and bacterial infection compared to viral infections (COVID-19, influenza, and other CoVs). This suggests shared molecular disruptions involving mitochondrial function and protein synthesis pathways between RIs and PD (Figure 5).

### 3.4. Co-Expression Modules

**Figure 6:**
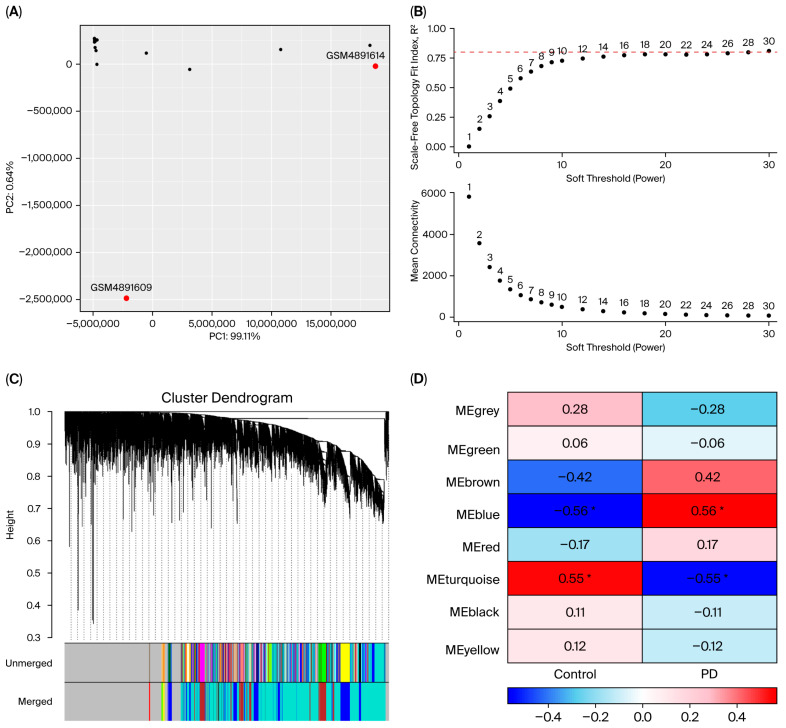
WGCNA results reveal PD-associated gene co-expression modules. (**A**) PCA identified two outlier samples (red), which were excluded from downstream analysis to ensure network robustness. (**B**) Determination of the soft-thresholding power. The scale-free topology fit index (top) indicated that R^2^ > 0.80 was achieved at power 18, which was selected as the optimal threshold. The corresponding mean connectivity (bottom) decreased with increasing power. The red dashed line is explained (R^2^ > 0.80). (**C**) Hierarchical clustering dendrogram of genes based on topological overlap matrix (TOM) dissimilarity. Distinct modules of co-expressed genes are represented by different colors. (**D**) Module–trait correlations between eigengenes and sample groups (control vs. PD). Red indicates positive correlations, and blue indicates negative correlations, with intensity reflecting correlation strength. Asterisks denote statistically significant associations: * *p* < 0.05. Notably, the MEturquoise module was positively correlated with control, while the MEblue and MEbrown modules showed strong negative correlations.

**Figure 7:**
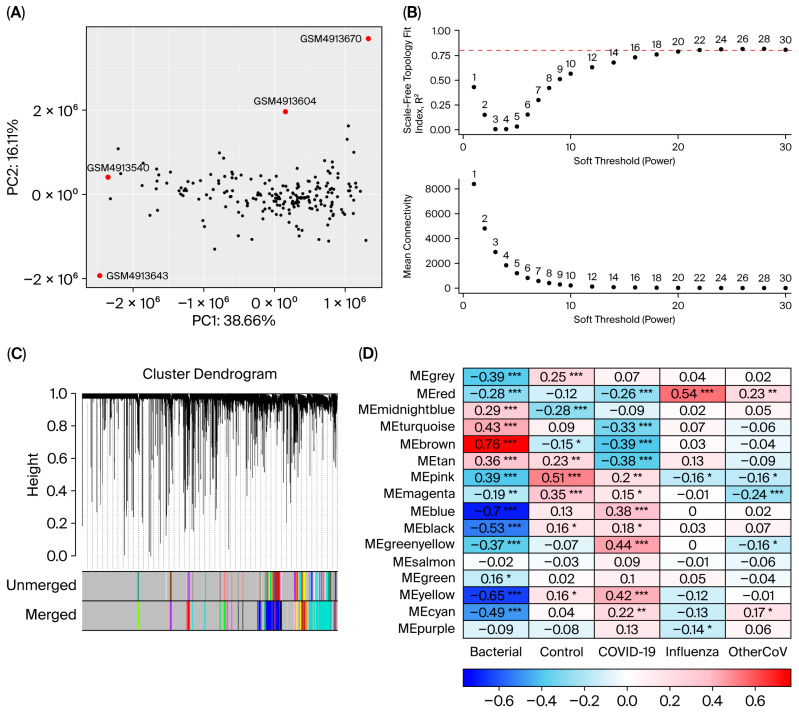
WGCNA results reveal RI-associated gene co-expression modules. (**A**) PCA identified four outlier samples (red), which were excluded from downstream analysis to ensure network robustness. (**B**) Determination of the soft-thresholding power. The scale-free topology fit index (top) indicated that R^2^ > 0.80 was achieved at power 20, which was selected as the optimal threshold. The corresponding mean connectivity (bottom) decreased with increasing power. The red dashed line is explained (R^2^ > 0.80). (**C**) Hierarchical clustering dendrogram of genes based on topological overlap matrix (TOM) dissimilarity. Distinct modules of co-expressed genes are represented by different colors. (**D**) Module–trait correlations between eigengenes and sample groups (control vs. RIs conditions). Red indicates positive correlations, and blue indicates negative correlations, with intensity reflecting correlation strength. Asterisks denote statistically significant associations: * *p* < 0.05, ** *p* < 0.01, *** *p* < 0.001. Notably, the MEpink module was positively correlated with control.

**Figure 8:**
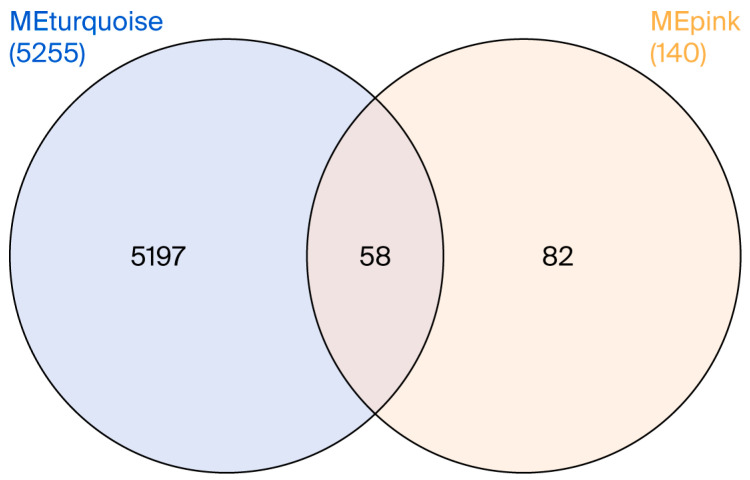
Overlap between MEturquoise and MEpink co-expression modules. Venn diagram showing the overlapped genes between the MEturquoise (MEturquoise, 5255 genes) and MEpink (MEpink, 140 genes) WGCNA modules. A total of 58 genes were shared between the two modules, while 5197 genes were unique to the MEturquoise module, and 82 were unique to the MEpink module. The shared genes may represent a transcriptional link between the two modules, suggesting partially overlapping biological functions.

Prior to performing WGCNA, PCA was applied to the normalized gene expression matrix to detect outlier samples. This quality control step is critical to enhance the reliability and accuracy of the analysis outcomes. PCA revealed two outlier samples (GSM4891609 and GSM4891614) in the PD dataset and four outlier samples (GSM4913540, GSM4913604, GSM4913643, and GSM4913670) in the RIs dataset, which were subsequently excluded (Figure 6 and Figure 7A). The soft-thresholding power was set to 20 and 18 for the RIs and PD datasets, respectively, based on the criteria of scale-free topology and mean connectivity. At this threshold, the scale-free topology fit index reached the recommended value of 0.8, ensuring robust network construction (Figure 6 and Figure 7B). After removing outlier genes and samples, a total of 15,648 genes from the RIs and 11,021 genes from the PD dataset were retained to construct module–trait relationships. Hierarchical clustering and the generation of module–trait correlation heatmaps led to the identification of 16 and 8 co-expression modules for RIs and PD, respectively (Figure 6 and Figure 7C). These modules comprised gene clusters that were either positively or negatively correlated with RIs and PD, with genes exhibiting both upregulation and downregulation patterns, indicating diverse transcriptional patterns in response to RIs and PD. The MEpink module exhibited the strongest positive correlation with the healthy group in the RIs dataset, while MEturquoise was significantly correlated with the healthy condition in PD dataset (Figure 6 and Figure 7D). In other words, these modules are correspondingly negatively correlated with RIs and PD, or, at most, they are only weakly positive. The MEpink and MEturquoise modules contained 140 and 5255 co-expressed genes, respectively. A total of 58 genes is common to both modules, suggesting potential transcriptomic convergence between PD and RIs (Figure 8). The overlapping genes between MEturquoise and MEpink modules are mentioned in (Table 4).

### 3.5. P-P Networks and HUB Genes

**Figure 9:**
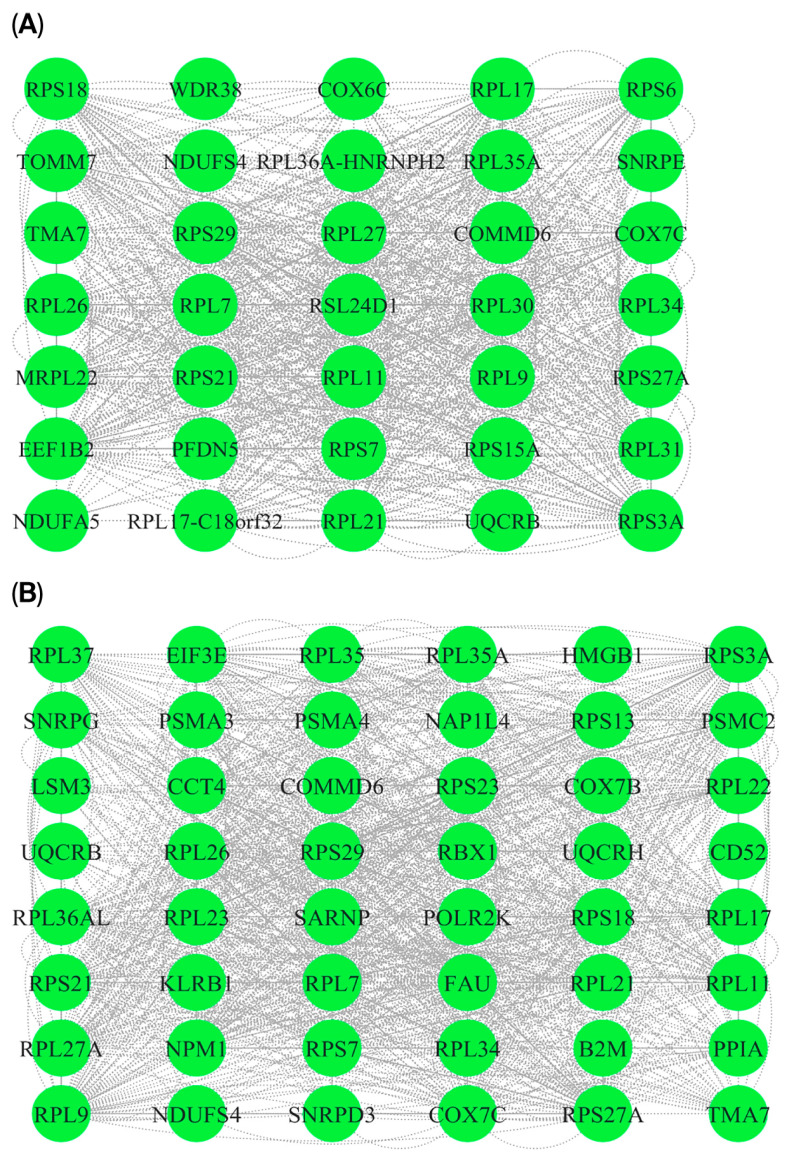
Protein–protein interaction (PPI) networks of overlapping genes. (**A**) PPI network of overlapping genes identified through DGE analysis. (**B**) PPI network of overlapping genes based on cross-module shared gene set, specifically from the MEpink module in RIs and the MEturquoise module in PD. Green nodes represent genes, and gray edges indicate experimentally validated or predicted protein–protein associations obtained from the STRING database. The dense interconnectivity highlights the strong functional association among ribosomal proteins, mitochondrial genes, and proteasome subunits, pointing to shared transcriptional patterns between RIs and PD.

**Figure 10:**
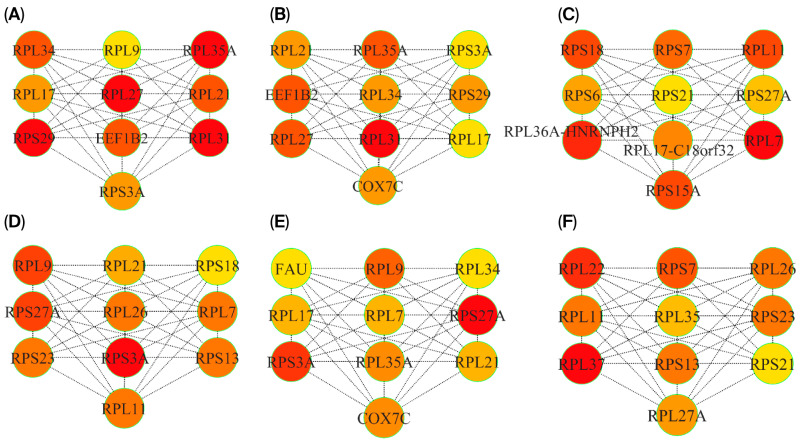
Hub genes identified from overlapping gene sets between PD and RIs using cytoHubba. (**A**–**C**) Top-ranked hub genes derived from overlapping DEGs, ranked using three network centrality algorithms: (**A**) Maximal Clique Centrality (MCC), (**B**) Maximum Neighborhood Component (MNC), and (**C**) Density of Maximum Neighborhood Component (DMNC). (**D**–**F**) Hub genes identified from overlapping WGCNA modules (MEpink in RIs and MEturquoise in PD), ranked using the same algorithms: (**D**) MCC, (**E**) MNC, and (**F**) DMNC. Node colors represent the degree of connectivity, with red indicating highly connected hub genes and yellow indicating lower connectivity. Most hub clusters are dominated by ribosomal proteins.

To investigate the molecular connections between PD and RIs, P-P interaction networks were constructed using genes that overlap between the two conditions. Two approaches were employed: (i) analysis of overlapping DEGs and (ii) considering cross-module overlapping genes identified via WGCNA. The DEG-based network comprises 40 nodes and 400 edges (Figure 9A), while the module-based network contains 54 nodes and 490 edges (Figure 9B). The DEG network features a densely connected core of ribosomal proteins such as (RPL7, RPS7, RPL11, RPS15A, RPS18), which may indicate translational stress. Mitochondrial genes such as (COX7C, TOMM7, NDUFS4, and UQCRB) are associated with mitochondrial function, suggesting potential overlap in mitochondrial disruption. Conversely, the network derived from the intermodule overlap gene set also includes genes enriched in ribosomal subunits and mitochondrial complex regulators but shows broader functional diversity. Key additions include immune-related genes (CD52, HMGB1) and proteasomal subunits (PSMA3, PSMA4, PSMC2). Together, these networks highlight potential overlaps in translational and mitochondrial stress pathways as shared mechanisms in both PD and RIs. The DEG-based network reflects direct translational changes, while the WGCNA- based network captures functionally coordinated modules, offering complementary insights into convergent pathobiology. To identify key regulatory genes shared between PD and RIs, the two networks were analyzed using CytoHubba. Hub gene ranking was based on three topological algorithms: Maximal Clique Centrality (MCC), Density of Maximum Neighborhood Component (DMNC), and Maximum Neighborhood Component (MNC) (Figure 10A–F). As a result of forming a major portion of the DEG-based network, ribosomal subunits consistently emerged across all three ranking methods. The recurrence of these genes suggests that ribosomal dysfunction and disrupted protein synthesis may represent common transcriptomic features observed in both PD and RIs. Additionally, EEF1B2, identified in the DEG-based network using the MNC-ranked algorithm, implicates RNA-binding and translational regulation processes relevant to both neurodegenerative and immune responses. Similarly, the hub genes from the overlapped co-expression modules shared prominently featured ribosomal proteins, emphasizing the involvement of the protein synthesis process. Interestingly, COX7C was also identified as a hub gene in the MNC-ranked network. As a component of the mitochondrial electron transport chain, COX7C is a component of the mitochondrial electron transport chain involved in ATP synthesis and has been dysregulated in both PD pathogenesis and host responses to RIs.

### 3.6. Functional and Pathway Enrichment Analysis

**Figure 11:**
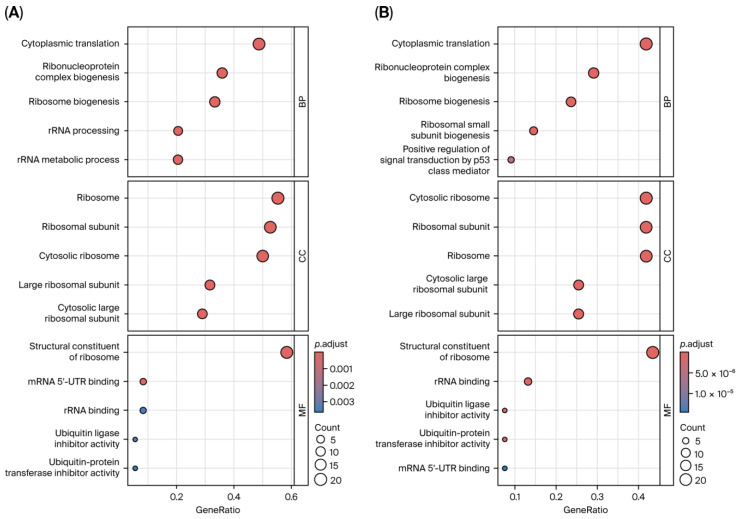
Functional enrichment of overlapping genes between PD and RIs. (**A**) Dot plot showing Gene Ontology (GO) enrichment analysis of shared DEGs between PD and RIs. (**B**) Dot plot showing GO enrichment results of intermodular overlap gene set (MEturquoise in PD and MEpink in RIs). Enriched terms are primarily associated with ribosome biogenesis, rRNA processing, cytoplasmic translation, and structural constituents of the ribosome, highlighting shared transcriptional perturbations in protein synthesis machinery. Circle size represents the number of genes enriched in each term, and color intensity corresponds to adjusted *p*-values.

**Figure 12:**
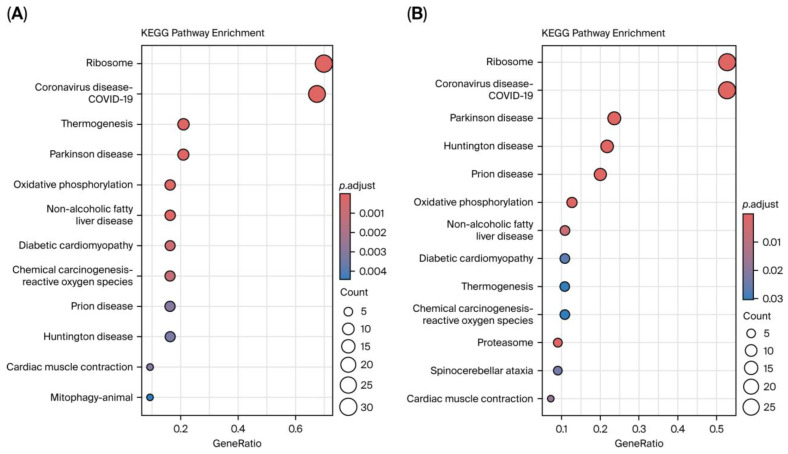
KEGG pathway enrichment of overlapping genes between PD and RIs. (**A**) Dot plot showing KEGG enrichment analysis of shared DEGs between PD and RIs. (**B**) Dot plot showing KEGG enrichment results of intermodular overlap gene set (MEturquoise in PD and MEpink in RIs). The enriched pathways highlight Ribosome, Oxidative phosphorylation, Proteasome, and neurodegenerative disease-related pathways (including PD, Huntington’s disease, and Prion diseases), indicating shared molecular disruptions in protein synthesis, mitochondrial function, and protein degradation. Circle size indicates the number of genes enriched in each pathway, and color represents adjusted *p*-values.

To explore the functional correlation between PD and RIs, Gene Ontology (GO) and KEGG enrichment analysis was conducted based on overlapping DEGs and shared genes from co-expression modules identified via WGCNA. Functional enrichment analysis showed that the PD–RI crosstalk DEGs were significantly enriched in GO:BPs such as cytoplasmic translation, ribonucleoprotein complex biogenesis, ribosome biogenesis, rRNA processing, and rRNA metabolism. GO:CC analysis indicated that the top enriched terms were exclusively associated with ribosomal structure, including ribosomal subunits, and cytosolic large ribosomal subunits. Within the GO:MF enrichment, the structural constituent of ribosomes was the most significant term, followed by mRNA 5′-UTR binding, rRNA binding, ubiquitin ligase inhibitor activity, and ubiquitin–protein transferase inhibitor activity (Figure 11A). The consistent appearance of ribosomal components and associated processes across multiple categories suggests that ribosomal dysregulation may reflect a common transcriptomic signature in both RIs and PD. Likewise, the shared gene set co-assigned to both WGCNA modules from PD and RIs exhibited similar enrichment patterns, highlighting the dominance of ribosome-related processes (Figure 11B). In overlapping DEGs, pathway enrichment using the KEGG database highlighted significant associations with several disease-related and metabolic pathways. Among these, Ribosome and Coronavirus disease COVID-19 were most significantly enriched, alongside pathways related to Thermogenesis, PD, and Oxidative phosphorylation (Figure 12A). Meanwhile, the overlapping genes identified across condition-specific WGCNA modules revealed stronger neurodegenerative signaling convergence. Similarly, the most significantly enriched pathways included Ribosome, Coronavirus disease (COVID-19), PD, Huntington’s disease, and Prion diseases, highlighting substantial convergence of neurodegeneration-related transcriptional processes. Consistent enrichment of ribosome, oxidative phosphorylation, and proteasome pathways indicates common disturbances in mitochondrial function and protein homeostasis across PD and RIs (Figure 12B).

### 3.7. Random Forest Model

**Figure 13:**
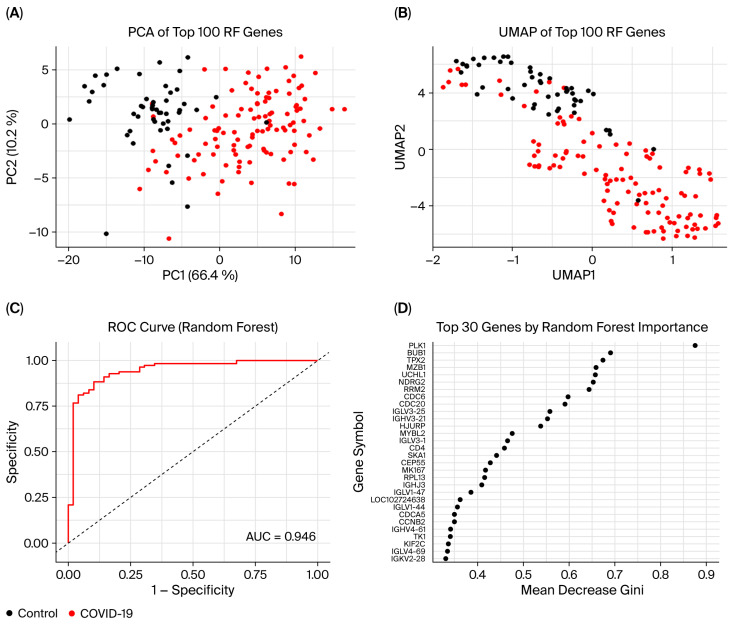
Performance and feature visualization of COVID-19 versus control Random Forest classifier using the top 100 genes. (**A**) PCA of the top 100 featured genes. Samples are colored by group (control = black; COVID-19 = red). (**B**) Uniform Manifold Approximation and Projection (UMAP) embedding of the same features, showing clear separation of control (black) and COVID-19 (red) samples in two dimensions. (**C**) Receiver operating characteristic (ROC) curve for the Random Forest model. The area under the curve (AUC) is 0.946, indicating high discriminative performance. (**D**) Variable importance plot displaying the top 30 genes by mean decrease in Gini impurity. Genes at the top contribute most to classification accuracy.

**Figure 14:**
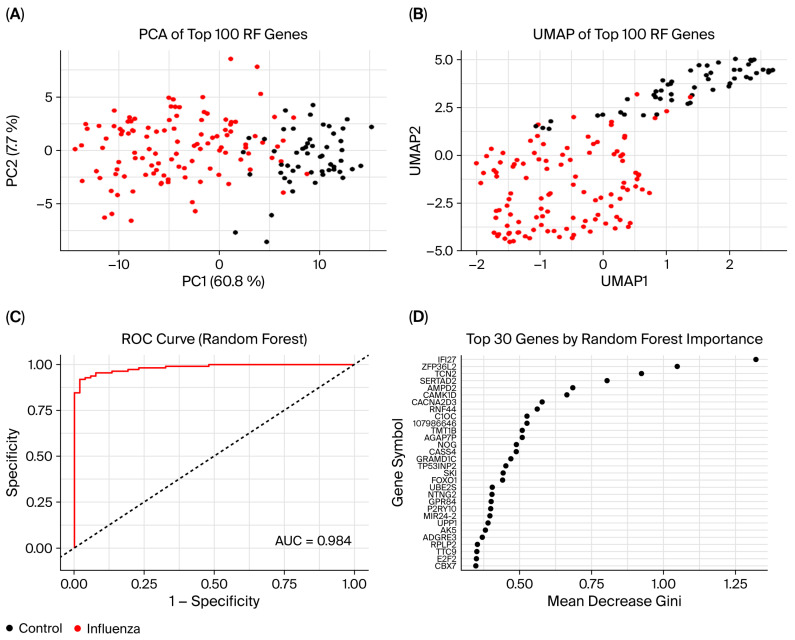
Performance and feature visualization of influenza versus control Random Forest classifier using the top 100 genes. (**A**) PCA of the top 100 featured genes. Samples are colored by group (control = black; influenza = red). (**B**) Uniform Manifold Approximation and Projection (UMAP) embedding of the same features, showing clear separation of control (black) and influenza (red) samples in two dimensions. (**C**) Receiver operating characteristic (ROC) curve for the Random Forest model. The area under the curve (AUC) is 0.984, indicating high discriminative performance. (**D**) Variable importance plot displaying the top 30 genes by mean decrease in Gini impurity. Genes at the top contribute most to classification accuracy.

To explore blood transcriptomic differences associated with RIs, we applied an RF model classifier to distinguish between COVID-19 and influenza versus healthy control samples using the top 100 most variable genes selected by the Mean Decrease Gini index. In the COVID-19 versus control model, the RF model classifier achieved high discriminative performance with an AUC of 0.946. PCA and uniform manifold approximation and projection (UMAP) both revealed explicit separation between COVID-19 and control samples, indicating clear separation in transcriptomic profiles (Figure 13A–C). The top-ranked genes contributing to model performance included PLK1, BUB1, TPX2, and MZB1, many of which are implicated in cell cycle regulation and immune signaling (Figure 13D). Similarly, in the influenza versus control model, the RF classifier achieved an even higher AUC of 0.984, achieving high classification performance. PCA and UMAP analysis again showed distinct clustering of infected and control samples (Figure 14A–C). The top-ranked genes for influenza classification included IFI27, ZFP36L1, TNC, SERTAD2, and AMPD3, several of which are known interferon-stimulated or antiviral response genes (Figure 14D). In both conditions, several ribosomal subunit genes were among the top features distinguishing COVID-19 or influenza from controls. In COVID-19, these included RPL13A, RPL6, RPL13, RPLP2, RPS11, UBA52, and the ribosomal pseudogene RPL13AP20. For influenza, discriminatory ribosomal genes included RPL7A, RPL13, RPL18, RPL23A, RPLP2, RPS2, and RPS4X. Additionally, genes linked to mitochondrial function and the UPS appeared less frequently among top-ranked features. In influenza, UBE2S, USP53, and TIMM10 were observed in this category, while in COVID-19, UBE2T, UBE2C, DTL, MT-ND2, UCHL1, and UBA52 were implicated.

## 4. Discussion

An increasing amount of evidence indicates that microbes with the ability to affect the respiratory system possess neurotropic activity. Virus-induced RIs such as those related to the influenza and SARS-CoV-2 have been epidemiologically and pathologically linked to cognitive deterioration and parkinsonism [50]. Similarly, a recent study found that severe pneumonia induces disruption of astrocytes and microglia functions and impairment of the blood–brain barrier by detecting the DNA segment of the pulmonary bacterial community in the brain [51]. Mitochondrial dysfunction, α-synuclein and Lewy pathology, and neuroinflammatory responses were suggested as potential pathways to elucidate host–microbial interactions in PD [52].

In the current study, we conducted blood-based transcriptomic analysis to reveal potential molecular connections between RIs and PD. The standard bioinformatics procedures were performed, and then, the results were validated using an RF model with external datasets. We found that PD and RIs partly follow similar transcriptional patterns. Evidently, many large and small ribosomal subunits, such as RPL11, RPS27, RPL21P28, RPS7, RPS27A, RPL31, RPL35A, RPL9, RPL34, RPS3A, RPS18, RPL23P8, RPL7, RBIS, RPL30, RPS6, RPL21, RPS29, RSL24D1, RPS15A, RPL26, RPL23, RPL17-C18orf32, RPL17, RPS21, RPL36A-HNRNPH2, and RPL36A, were found to be differentially downregulated under both conditions. WGCNA suggested that these proteins may act in a coordinated manner and are associated with both RIs and PD. This finding is consistent with two recently published studies; one conducted an in-depth proteomic analysis of the substantia nigra tissue from individuals diagnosed with PD. The findings revealed a significant downregulation of various mitochondrial and ribosomal proteins, indicating a disruption in the protein synthesis machinery within dopaminergic neurons. The other study performed transcriptomic analysis for PD samples and reached the same conclusion [53,54]. In the same context, several previous studies have shown that multiple ribosomal subunits, including RPL5, RPL6, RPL11, RPL23, RPL26, RPL37, RPS7, RPS14, RPS15, RPS20, RPS25, RPS26, RPS27, RPS27A, RPS27L, RPS3, RPS6, RPS19, RPL13A, and RPL22, play essential roles in regulating inflammation and cell proliferation. These ribosomal subunits function in synchronization and have a crucial impact irrespective of their conventional role in protein synthesis [55,56,57]. Major cellular processes like morphogenesis, hemopoiesis, inflammation, tumorigenesis, and proteostasis have been demonstrated to be under the control of various ribosomal subunits [58,59,60]. The ribosomal subunits are involved in antiviral immunity-related signaling pathways, including the NF-κB and MAPK pathways. Additionally, they act as a double-edged sword: they can interfere with viral molecules, or in some cases, they mediate viral pathogenicity by promoting the viral molecules to enter and replicate into the host cells [61]. Multiple respiratory pathogens including influenza and SARS-CoV-2 viruses are reported to cause ribosomal stress and impairment of ribosomal biogenesis via different mechanisms [62,63]. KEGG enrichment analysis of the dysregulated genes found several neurological disease pathways that were significantly enriched, including pathways in Prion diseases, PD, and Huntington’s disease. The “Ribosome” pathway was the most enriched term, indicating the possible implication of impaired ribosomal biogenesis in pneumonia-related neurodegeneration. Various ribosomal proteins have been reported to directly interact with Mdm2, repressing E3 ubiquitin ligase activity to stabilize and activate the oncoprotein of p53. This RP–Mdm2–p53 pathway operates as an essential cellular stress response network that connects defective ribosome biogenesis to p53-dependent cell cycle arrest or apoptosis. Under normal conditions, Mdm2 represses p53 levels by promoting its ubiquitin-dependent degradation; however, upon ribosomal stress, ribosomal proteins, including RPL11 and RPL5, interact with Mdm2 and block p53 degradation, providing a protective response to cellular damage [64,65]. Ribosomal stress induced by Zika virus leads to elevated p53 levels by facilitating a connection between RPL11 and MDM2, ultimately encouraging apoptosis in neuronal cells. This indicates that the RPL11-MDM2-p53 pathway likely plays a role in the neuropathogenic effects of this virus [66]. In chronic neurodegenerative diseases, apoptosis is the main type of cell death. The death of one cell can impact the cell death process in nearby cells. Factors released by dying and post-dying cells can be harmful to neighboring cells. Nearby cells are exposed to stimuli that are analogous to those affecting dying cells [67]. These observations suggest that dysregulated ribosomal pathways could potentially influence the RP–MDM2–P53 stress response and apoptosis. The high level of p53 is double-edged sword, as it can be beneficial to eliminate damaged cells but detrimental in some cases, particularly if healthy cells are affected.

The most interesting downregulated ribosomal subunit is RPS27A. Ribosomal protein S27a (RPS27A) is a versatile ribosomal protein that plays a role in ribosome biogenesis and protein synthesis. RPS27A is also expressed together with ubiquitin as fusion proteins; however, they operate independently once ubiquitin is separated from RPS27A by deubiquitinating enzymes. As a result, RPS27A is involved in independent post-translational protein modifications by regulating ubiquitylation [68,69]. Recently, research found RPS27A was identified as a regulatory gene that could induce microglial activation by regulating cytokine-mediated signaling cascades, thereby contributing to neuroinflammation and progression of neurological diseases [70]. Another study found that RPS27A drives neuroinflammation in cerebral ischemia–reperfusion injury via the NF-κB signaling pathway [71].

Additionally, transcriptomic overlaps indicate that RIs and PD share alterations in UPS- and autophagy-related genes, suggesting a possible contribution of these pathways to disrupted protein homeostasis. DGE analysis revealed that RPS27A, which encodes monoubiquitin, is dysregulated during different RIs cases. UPS and autophagy are involved in protein degradation; the initiation of these processes relies on the ubiquitin reservoir within cells. Both degradation systems utilize ubiquitin as a signal for degradation of substrates; the proteasome itself also could be subject to regulation and can be directed for autophagic degradation through ubiquitination [72,73]. In addition to RPS27A, WGCNA has exhibited several UPS-related genes that are negatively associated with RIs and PD, including PSMA3, PSMA4, and PSMC2. The proteasome is a large multi-subunit complex present in both the nucleus and cytoplasm responsible for proteolysis [74]. During neurodegenerative diseases, the functional efficiency of the UPS progressively declines, leading to proteotoxic stress. Failure in eliminating toxic misfolded proteins has been observed in several neurological disorders, with mutant huntingtin in Huntington’s disease, α-synuclein in PD, mutant SOD1 in familial amyotrophic lateral sclerosis, and β-amyloid in Alzheimer’s disease representing optimal examples of the relationship between proteasome malfunction and neurodegeneration [75]. While the UPS exerts dual pro-viral and anti-viral pathophysiological effects during infection. It enhances viral protein function and stability while also mediating degradation of viral components as a host defense mechanism [76]. It was also found that the presence of a fully operational UPS is essential for the effective eradication of intracellular *Streptococcus pneumoniae* in brain endothelial cells [77].

Moreover, DGE analysis revealed that several genes encoding subunits of mitochondrial complexes involved in the electron transport chain and ATP production, including NDUFS4, NDUFA5, COX6C, COX7C, and UQCRB, were downregulated under both PD and RIs conditions. WGCNA modules supported this observation via the identification of overlapping and additional electron transport chain-related genes (UQCRH, NDUFS4, COX7C, UQCRB, and COX7B) that showed negative associations with RIs. Emerging evidence indicates that mitochondrial dysfunction represents a central pathogenic feature of PD, integrating the impact of genetic mutations such as PINK1, PRKN, and LRRK2 and environmental exposure to neurotoxins such as Rotenone and MPTP. These factors impair mitochondrial quality control, disrupt oxidative phosphorylation, and promote oxidative stress, collectively driving dopaminergic neurodegeneration [78]. Despite the obvious relevance of mitochondria in neuronal bioenergetics, they perform various additional functions. Among these is the regulation of Ca^2+^ levels, a crucial process that could be particularly vital in the axons of certain neurons. Additionally, mitochondria play a key role in metabolic signaling as essential providers of citrate, a critical component for the synthesis of acetyl-coenzyme A, as well as protein and DNA acetylation [79]. Despite their relatively contradictory outcomes, various studies have investigated the role of mitochondria during infections, and multiple studies have reported downregulation of mitochondrial complexes subunits during COVID-19 and influenza infection. These variations could be attributed to disease progression and the types of implicated complexes [80]. Mitochondria serve as central hubs in innate immune signaling and apoptosis during infection. Given the fundamental role of mitochondria in cellular regulation, their involvement in host responses to infection is well established. Numerous studies demonstrate that pathogens subvert mitochondrial function to promote intracellular survival, facilitate dissemination, induce mitochondria-mediated cell death, or evade immune responses [81].

This study has several limitations that should be acknowledged. First, because the analyses were based on publicly available datasets, detailed metadata such as age, sex, comorbidities, and medication history were not consistently available, preventing adjustment for these potential confounding factors. Second, heterogeneity among the included respiratory infection datasets, including differences in sample sizes, sequencing platforms, and clinical characteristics, may have introduced variability and influenced the generalizability of our findings. Third, this study relied exclusively on secondary transcriptomic data, which limits the ability to draw causal inferences. Future work using larger, clinically homogeneous cohorts with detailed annotations, combined with experimental validation, will be necessary to confirm and extend these findings.

## Figures and Tables

**Table 1:** Summary of RNA-seq datasets utilized in this study. The table provides the GEO accession numbers, sequencing platforms, sample sources, sample sizes, and analyses performed. Only the samples included in the present study are listed. Detailed demographic information (e.g., age, sex) was not uniformly available across all datasets.

GEO Accession	Platform	Source	Samples	Analysis	Publishing Year
GSE161731	Illumina NovaSeq 6000	Blood	COVID-19 (*n* = 77)	DGE and WGCNA	2021
			B. pneumonia (*n* = 24)		
			Seasonal coronaviruses (*n* = 61)		
			Influenza (*n* = 17)		
			Control (*n* = 19)		
GSE161199	Illumina NextSeq 500	Blood	PD (*n* = 5)	DGE and WGCNA	2020
			Control (*n* = 11)		
GSE196350	Illumina HiSeq 4000	Blood	Influenza (*n* = 33)	RF	2024
			Control (*n* = 16)		
GSE155635	Illumina HiSeq 4000	Blood	Influenza (*n* = 60)	RF	2024
			Control (*n* = 16)		
GSE213168	Illumina NovaSeq 6000	Blood	Influenza (*n* = 18)	RF	2024
			Control (*n* = 20)		
GSE171110	Illumina HiSeq 2500	Blood	COVID-19 (*n* = 44)	RF	2021
			Control (*n* = 10)		
GSE167000	Illumina NovaSeq 6000	Blood	COVID-19 (*n* = 51)	RF	2021
			Control (*n* = 22)		
GSE152418	Illumina NovaSeq 6000	Blood	COVID-19 (*n* = 16)	RF	2020
			Control (*n* = 17)		

**Table 2:** The total number of DEGs and the counts of significantly upregulated and downregulated transcripts across RIs and PD.

Condition	Database	DEGs	Upregulated	Downregulated
COVID-19	GSE161731	812	327	485
Influenza		1395	812	583
OtherCoV		934	497	437
B. pneumonia		6769	2465	4304
PD	GSE161199	2479	304	2175

**Table 3:** Shared DEGs across RIs and PD. The first row lists genes that are significantly differentially expressed in all four RIs. The second row shows the subset of these “Shared RIs DEGs” that also appear as DEGs in PD.

Shared RIs DEGs	HES4, RPL11, CD52, MAN1A2, RPS27, GAS5-AS1, GAS5, SNORA103, MIR4426, SNRPE, RPL21P28, RPS7, RPS27A, SNRPG, RPL31, DBI, LINC01934, ANKRD44-AS1, PPIL3, EEF1B2, LSM3, TMA7, MIX23, NMRAL2P, RPL35A, RPL9, BDH2, RPL34, SNHG8, RPS3A, NDUFS4, COX7C, HINT1, MRPL22, RPL26L1, RPS18, RPS10-NUDT3, RPS10, NDUFA4, RPL23P8, TOMM7, SEM1, NDUFA5, RPL7, RBIS, UQCRB, RPL30, COX6C, LY6E, RPS6, TOMM5, GKAP1, WDR38, RPS24, KIF20B, IFITM3, KLRB1, LOC107987174, KLRF1, KLRC4, KLRC2, PFDN5, MYG1-AS1, SARNP, RPL41, RPL6, RPL21, CCNA1, TPT1, COMMD6, RPS29, ATP5MJ, RSL24D1, RPS17, CPEB1, RPS15A, RPL26, SNORD3A, LOC107984974, RPL23, RPL27, RPL17-C18orf32, RPL17, SNORD58B, ZNF85, ZNF302, HPN, TRAPPC2B, RPS21, FAM247A, RBFOX2, RBX1, COX7B, RPL36A-HNRNPH2, RPL36A, RPL39, SH2D1A, FGF13
Overlapping DEGs between RIs and PD	HES4, RPL11, GAS5, SNORA103, MIR4426, SNRPE, RPL21P28, RPS7, RPS27A, RPL31, EEF1B2, TMA7, RPL35A, RPL9, RPL34, SNHG8, RPS3A, NDUFS4, COX7C, MRPL22, RPS18, RPL23P8, TOMM7, NDUFA5, RPL7, RBIS, UQCRB, RPL30, COX6C, RPS6, WDR38, KIF20B, KLRB1, PFDN5, MYG1-AS1, RPL21, COMMD6, RPS29, RSL24D1, RPS15A, RPL26, SNORD3A, RPL27, RPL17-C18orf32, RPL17, SNORD58B, ZNF302, RPS21, RPL36A-HNRNPH2

**Table 4:** List of genes overlapping between the MEturquoise and MEpink co-expression modules identified by weighted gene co-expression network analysis (WGCNA).

Overlapped genes between MEturquoise (PD) and MEpink (RIs) modules	RPL22, RPL11, UBXN11, CD52, UQCRH, UFC1, RPS7, RPS27A, CCT4, SNRPG, DBI, LSM3, TMA7, CSTA, RPL35A, RPL9, RPL34, RPS3A, RPL37, NDUFS4, RPS23, COX7C, NPM1, RPS18, PPIA, PSMC2, RPL7, UQCRB, POLR2K, EIF3E, ATP6V1G1, RPL35, NAP1L4, RPL27A, RPS13, FAU, KLRB1, LDHB, SARNP, RPL21, HMGB1, COMMD6, RPS29, RPL36AL, PSMA3, ATP5MJ, B2M, PSMA4, RPL26, SNHG29, RPL23, RPL17, RPS21, SNRPD3, RBX1, COX7B, MIR10393, MYG1-AS1

## Data Availability

The RNA-seq datasets analyzed in this study are publicly available in the NCBI Gene Expression Omnibus (GEO) repository. Additional processed data generated during this study are available from the corresponding author upon reasonable request.

## Abbreviations

The following abbreviations are used in this manuscript:

PD	Parkinson’s disease
RIs	Respiratory infections
DEGs	Differentially expressed genes
DGE	Differential gene expression
PCA	Principle component analysis
GO	Gene ontology
RF	Random forest
CNS	Central nervus system
WGCNA	Weighted gene co-expression network analysis
PPI	Protein-protein interaction
